# The urinary microbiome distinguishes symptomatic urinary tract infection from asymptomatic older adult patients presenting to the emergency department

**DOI:** 10.1080/21505594.2025.2546063

**Published:** 2025-08-09

**Authors:** Evan S. Bradley, Celina Stansky, Abigail L. Zeamer, Ziyuan Huang, Lindsey Cincotta, Abigail Lopes, Linda Potter, Theresa Fontes, Doyle V. Ward, Vanni Bucci, Beth A. McCormick, John P. Haran

**Affiliations:** a Department of Emergency Medicine, UMass Memorial Medical Center, Worcester, MA, USA; b Program in Microbiome Dynamics, UMass Chan Medical School, Worcester, MA, USA; c Department of Microbiology, UMass Chan Medical School, Worcester, MA, USA; d Clinical Renal Laboratory, UMass Memorial Medical Center, Worcester, MA, USA

**Keywords:** Urinary tract infection, metagenomic analysis, machine learning, older adults, emergency department, asymptomatic bacteriuria

## Abstract

Older adults suffer from a high rate of asymptomatic bacteriuria (ASB), in which urinalysis may appear positive (presence of bacteria, white blood cells, and nitrates), often triggering initiation of antibiotics in acute care settings, without actual urinary tract infection (UTI) present. To investigate the urinary microbiome of older adults being tested for UTI, we enrolled a convenience sample of 250 older adult Emergency Department patients who had microscopic urinalysis ordered as part of their routine clinical care. Urinalysis results were classified as positive or negative, and patients were classified as being symptomatic or asymptomatic based on established diagnostic guidelines. We sought to determine if features of the urinary microbiome differed between positive and negative urinalysis (UAs) and symptomatic and asymptomatic patients with positive UAs. The same urine sample used for clinical testing was sequenced and analyzed for bacterial taxa, metabolic pathways, and known bacterial virulence factors. After exclusion of anatomical abnormalities and filtering for sequencing quality, 152 samples were analyzed (5 negative UAs, 147 positive UAs, among which 68 were asymptomatic, and 79 symptomatic). Positive UA samples showed significantly lower alpha diversity (2.29 versus 0.086, *p* < 0.01) and distinct community composition based on beta-diversity (PERMANOVA on Bray-Curtis distance *p* < 0.01). Alpha and beta diversity did not significantly differ between asymptomatic and symptomatic patients. Machine learning classifiers combining clinical covariates other than specific signs and symptoms and microbiome features (taxa, metabolic pathways, or virulence factors) revealed mostly microbiome features as predictive of symptomatic UTI over clinical features.

## Introduction

It was once thought that human urinary tracts were normally sterile environments, but it is now known that there is a community of microorganisms that resides in the urinary tract, known as the urinary microbiome or uromicrobiome [1–8]. Changes in the urinary microbiome have been connected to a wide variety of genitourinary conditions such as urinary urge incontinence, pelvic pain syndromes, and recurrent urinary tract infections (UTIs) [9,10]. Annually in the US, UTI is listed as the cause of some 1 million Emergency Department (ED) visits and 15% of acute care hospitalizations among patients 65 years of age and older [11]. UTI is a potentially life-threatening diagnosis as it can progress to sepsis and septic shock, which carries high mortality [12]. Despite its prevalence and impact on the healthcare system, appropriate diagnosis of UTIs remains a challenge, especially in older adults. This is because a substantial portion of the population over age 65 have a condition known as asymptomatic bacteriuria (ASB) in which the urinary tract is colonized with potentially pathogenic bacteria, but the individual does not have symptoms and does not benefit from treatment with antibiotics [13]. ASB is defined by the Infectious Disease Society of America (IDSA) as a urine culture growing 10^5^ colony forming units of a known urinary pathogen from an individual who is having no symptoms [14]. Since urine cultures can take up to 3 days, what is typically relied upon as a proxy in the ED are the results of microscopic urinalysis (UA) that are suggestive of infection, i.e. the presence of nitrites, the presence of leukocyte esterase greater then trace, or greater than 10 white blood cells per high-powered field on microscopy [15]. The IDSA only recommends treating these patients with antibiotics only if they have signs and symptoms of a urinary tract infection such as dysuria, lower abdominal or perineal pain in the case of uncomplicated infection, or flank pain and fever in the case of complicated infection, coincident with UA findings that are suggestive of infection [14]. Guidance from the IDSA suggests that, except for pregnant women or those undergoing invasive urologic procedures, there is no benefit to treating ASB with antibiotics [16]. ASB prevalence is 10–20% in community-dwelling older adults [17] and as high as 50% in those who reside in skilled nursing facilities [13]. Among patients with positive urine cultures admitted to an inpatient service from the ED about 25% did not have signs or symptoms of UTI [18]. These discrepancies highlight the difficulties in accurately distinguishing ASB from UTI, especially in the acute care setting.

The clinical tests most frequently used to diagnose UTI, the UA and urine culture, have significant limitations. A study of adult ED patients suggested that a UA with features previously mentioned as associated with UTI had a specificity of 54% and a positive predictive value of 38% for a positive urine culture [15]. Because the UA alone lacks specificity for diagnosis of UTI, published guidelines suggest that UA should only be used in patients with signs and symptoms of UTI [19,20]. Despite this, clinicians frequently test for and initiate treatment for presumed UTI in the absence of signs or symptoms of infection [21]. A study of patients admitted from the ED to a general medical service found that as many as 80% of older patients had a UA ordered without the presence of urinary symptoms [22]. This discrepancy between actual practice and recommended guidelines may in part reflect the poor specificity of current standard diagnostic procedures as well as the desire of physicians to be thorough when presented with cases with multiple possible differential diagnoses, as is often the case in the ED setting.

The urinary microbiome may hold insights in distinguishing between ASB and UTI. There may be characteristics of the overall community that are suggestive of one state versus the other. An imbalance of pathogens over other commensal species in urine may be the microbiologic signature of shifting from ASB to UTI. Metagenomic data about the presence of specific virulence factor genes or metabolic pathways could provide additional clues. For example, Uropathogenic *Escherichia coli* (UPEC) typically require pathogenicity factors, often encoded on pathogenicity islands [23] to cause active disease; colonizing organisms in patients with ASB may lack genes for pathogenicity factors and thus, the presence or increased abundance of specific genes may be a reliable signature of UTI. There are also likely metabolic requirements for organisms to establish infection in the urinary tract that could signal a transition to UTI. Given that the urinary microbiome field is relatively new and comprehensive characterization is emerging, more studies, especially in clinical populations, are warranted.

The primary objective of this study was to examine the urinary microbiome of older adult patients with suspected UTI on initial evaluation in the ED. Using metagenomic sequencing and machine learning-based multimodal analysis we sought to determine if urinary microbiome features showed reliable differences between symptomatic versus asymptomatic patients, with the goal of selecting highly discriminatory features that could potentially be investigated as diagnostic targets.

## Materials and methods

### Patient recruitment

We enrolled in a convenience sample of adult patients over 65 years old admitted to the UMass Memorial University Campus ED from January 2022 through April 2023 who had a urinalysis ordered and collected as part of routine clinical care. Written informed consent was obtained from all patients. If a patient was deemed not to have capacity by medical providers or documented in their chart, written informed consent was obtained from patient’s legally authorized representative (next of kin or designated health care proxy). Those with potentially confounding anatomic abnormalities (e.g. urostomy or nephrostomy) and chronic indwelling Foley catheters on ED admission were excluded as these conditions may cause unknown long-term changes to the urinary microbiome not reflective of ASB. Patients who had a urinary catheter placed at the ED visit in which they were enrolled were not excluded, as this is frequently performed to obtain specimens from individuals who are incontinent of urine or otherwise cannot provide a sample on demand and represents an important population to study with respect to UTI. Patients were interviewed for current urinary symptoms including new onset dysuria (pain on urination), urinary frequency, urinary urgency, urinary incontinence, hematuria, urinary retention, lower abdominal pain, fever, or flank pain. Signs of urinary tract infection including lower abdominal tenderness, altered mental status, and costovertebral angle tenderness were obtained from electronic medical records. Patients were also asked about recent medical history including hospitalizations, surgeries, urinary catheterization, recent antibiotic treatment, and recent urinary tract infections. Past medical history, current medications, ED disposition, final diagnosis were all obtained via chart review. A complete list of patient variables that were collected and used as covariates within the models is shown in Table S3. We also review study participants’ medical charts up to 14 days post admission to determine if there were any additional unplanned hospitalizations, treatments for urinary tract infection, or death.

Urine samples were obtained by nursing staff in the ED either by mid-stream clean catch or urinary catheters in patients incontinent of urine or otherwise not able to provide a sample. Although as previously stated, anyone with a chronic indwelling urinary catheter on admission was excluded. Patients were categorized as having symptoms of UTI based on current diagnostic guidelines of signs and symptoms provided by IDSA and the Massachusetts Department of Public Health [13,19,20]. That is, the presence of dysuria considered most specific for UTI, or the presence of at least two signs or symptoms that could be attributed to UTI based on patient interview and physician chart review. These included fever, altered mental status, generalized weakness, lower abdominal pain, urinary frequency, urinary urgency, new urinary incontinence, new urinary retention, flank pain, or flank tenderness. Traditional urinalysis results were considered positive if there was at least one feature consistent with infection that is usually used by emergency physicians to make treatment decisions regarding UTI; these were the presence of nitrites, the presence of leukocyte esterase greater then trace, or greater than 10 white blood cells per high-powered field on microscopy [15]. The human subjects research protocol used for this study was approved by the UMass Chan Institutional Review Board (Docket # H00014312)

### Sample collection and processing

The remainder of the urine samples from enrolled patients provided to the clinical laboratory for traditional microscopic urinalysis and urine culture were kept at 4°C in the UMass Memorial Renal Laboratory until they were collected by study staff and then stored long term at −80°C. Samples were thawed and batch processed for sequencing in sets of 20 samples at a time. A total volume of 5 mL of each urine sample was used for metagenomic analysis. Left-over samples of less than 5 mL of urine were diluted with DNA/RNA shield solution (Zymo Research) to 5 mL each. Eukaryotic cell contaminants were first removed by low-speed spin at 180 g ×15 min at 4°C. Supernatants were then pelleted for bacteria by spinning at 3000 rcf ×15 min and washed once with 5 mL of DNA/RNA shield solution. The final pellet was resuspended in 250 µL of DNA/RNA shield and total DNA extracted using the Zymo Research DNA miniprep kit with bead-beating step. Samples were prepared for paired-end sequencing using the Nextera XT DNA library preparation kit (Illumina) and sequencing was performed on the Illumina NextSeq at the UMass Chan Center for Microbiome Research. Negative controls were not included in library preparation and sequencing and corrections for batch sequencing were not performed.

### Sequence processing and analysis

Shotgun metagenomic reads were first trimmed and quality filtered to remove sequencing adapters and host contamination using Trimmomatic [24] and Bowtie2 [25], respectively, as part of the KneadData pipeline version 0.10.0 (https://huttenhower.sph.harvard.edu/kneaddata/). Recognizing that urine samples have lower biomass, we have taken steps to ensure results would not be affected by random contamination by rejecting samples below a threshold of a certain number of metagenomics reads (500,000, see below). This helps ensure removal of samples representing low abundance laboratory contaminants and robust sequence counts for downstream analysis to reduce batch-to-batch variation.

Relative abundances of different microbial taxa were calculated using Metaphlan3 [26]. Relative abundances of detected microbial metabolic pathways were determined using HUMAnN3 [27] and measured as Read Per Kilobase Million (RPKM) (number of aligned reads/kilobase gene size * 1000000) to account for varying sizes of alignment targets. Relative abundances of virulence factors were measured by aligning KneadData processed reads against protein sequence databases of known bacterial virulence factors (http://www.mgc.ac.cn/VFs/) [28]. Sequences were aligned using the DIAMOND aligner [29] in blastx mode with default settings for sensitivity of alignment ( > 60% identity). Abundance of virulence factor genes was also measured in RPKM.

### Data analysis

Analyses were performed in R and Python. Shannon diversity indices, PERMANOVAs on Bray-Curtis distances, and tests of homogeneity of betadispersion were calculated using the vegan package [30]. Non-metric MultiDimensional Scaling (NMDS) ordination plots were generated using the R phyloseq package [31]. Differential abundance of taxonomies across variables of interest were measured with Maaslin2 [32], using the compound-Poisson linear model method due to the zero-inflated nature of urinary microbiomes and default settings for statistical significance and the Benjamini-Hochberg correction for multiple comparisons. Basic cleaning of collected clinical data was performed in python JupyterNotebook with the pandas and numpy packages. Specifically, unstructured clinical data was filtered for relevant clinical data of interest (e.g. diagnoses and chart notes directly associated with UTI) and objective variables were converted to binary for machine learning analyses. Urinary symptoms were left out of machine learning models as these were used to distinguish between asymptomatic patients with positive UA (suggestive of ASB) and symptomatic patients (suggestive of UTI) in our study; thus, inclusion would have introduced obvious bias into the models. Graphs were generated using the R ggplot2 package [33]. Final figures were arranged using Adobe Illustrator and BioRender.

### Machine learning based classifier analysis

We examined seven approaches for handling high-dimensional tabular datasets that have previously been applied to microbiome data, as well as a neural network-based algorithm, which can show superior performance with large datasets [34]: Elastic Net Regression [35], Least Absolute Shrinkage and Selection Operator (LASSO) regression [36], Ridge Regression [37], Support Vector Machine (SVM) regression [38], a Random Forrest Classifier [39] (RFC), TabNet [40], and XGBoost [41]. All approaches were implemented to predict symptomatic versus asymptomatic cases (“0” represents asymptomatic cases, and “1” represents symptomatic cases), utilizing features derived from clinical covariates which included the results of clinical testing, including the results of microscopic UA as well as pertinent past medical history listed in Table 1. This data was then combined with the results of microbiome analysis which included measurements of taxonomies, metabolic pathways, and known virulence factors represented in the sequencing data. This resulted in three datasets taxonomies + clinical covariates (761 features), metabolic pathways + clinical covariates (456 features), and virulence factors + clinical covariates (2171 features).

**Table 1.** Baseline study cohort demographics.

	Asymptomatic Patients	Symptomatic Patients	p
n	68	79	
Age (median [IQR])	80.00 [72.00, 87.25]	79.00 [73.00, 84.00]	0.408
Female (%)	56 (82.4)	61 (77.2)	0.572
White (%)	63 (92.6)	72 (91.1)	0.975
Black (%)	1 (1.5)	3 (3.8)	0.722
Other (%)	4 (5.9)	4 (5.1)	1
Ethnicity Hispanic (%)	4 (5.9)	2 (2.5)	0.545
Antibiotic Treatment in previous 90 days (%)	27 (39.7)	27 (34.2)	0.602
Treated in hospital with antibiotics for UTI (%)	57 (83.8)	66 (83.5)	1
Urinary Catheterization within 90 days (%)	5 (7.4)	11 (13.9)	0.313
Hospitalization within 90 days (%)	28 (41.2)	22 (27.8)	0.127
Resides in Private Home (%)	57 (83.8)	68 (86.1)	0.881
Resides in Assisted Living (%)	6 (8.8)	4 (5.1)	0.566
Resides in Nursing Home (%)	5 (7.4)	7 (8.9)	0.975
Surgery within 90 Days (%)	10 (14.7)	14 (17.7)	0.788
No Diagnosis of Dementia (%)	54 (79.4)	69 (87.3)	0.283
History of Alzheimer’s Dementia (%)	1 (1.5)	3 (3.8)	0.722
History of Vascular Dementia (%)	2 (2.9)	0 (0.0)	0.412
History of Dementia Not Otherwise Specified (%)	9 (13.2)	6 (7.6)	0.394
Baseline Incontinence of Urine (%)	32 (47.1)	40 (50.6)	0.79
History of Diabetes (%)	30 (44.1)	27 (34.2)	0.288
Active Cancer Diagnosis (%)	15 (22.1)	29 (36.7)	0.08
On Immunosuppressive Medication (%)	6 (8.8)	8 (10.1)	1
History of COPD (%)	7 (10.3)	23 (29.1)	0.009*
Baseline Incontinence of Stool (%)	7 (10.3)	12 (15.2)	0.525
Admitted to Hospital (%)	44 (64.7)	49 (62.0)	0.869
Placed in Observation (%)	22 (32.4)	23 (29.1)	0.806
Discharged from ED (%)	2 (2.9)	7 (8.9)	0.251

For in-depth explanations of the machine-learning based methods used in this analysis, please see our materials supplement. We examined model performances by F1 score, Area Under the Receiver-Operator Curve (AUC), Accuracy, Precision, Recall, and Specificity. The F1-score is a harmonic mean between precision and recall. This accounts for both prediction errors and the specific type of prediction error. We highlight the F1-score as our main model evaluation metric because the F1-score accounts for imbalanced data and extremes in either recall or precision while maximizing both. Thus, this metric is well suited for cases where both false positives and false negatives are undesirable. AUC provides a balanced measure between the true positive rate and false positive rate, describing how well a model distinguishes between positive and negative classes. Recall is the rate of true positive prediction. Precision is how often a true positive result is predicted. Specificity is how often a true negative result is predicted. Accuracy measures the proportion of both true positives and true negatives among the total number of cases, assessing the model’s overall correctness. F1 score identifies positive cases while equally penalizing false positives (FP) and false negatives (FN), thereby balancing concern for both precision and recall. A summary of these metrics for each of the different models is shown in Table S2.

## Results

**Figure 1.**
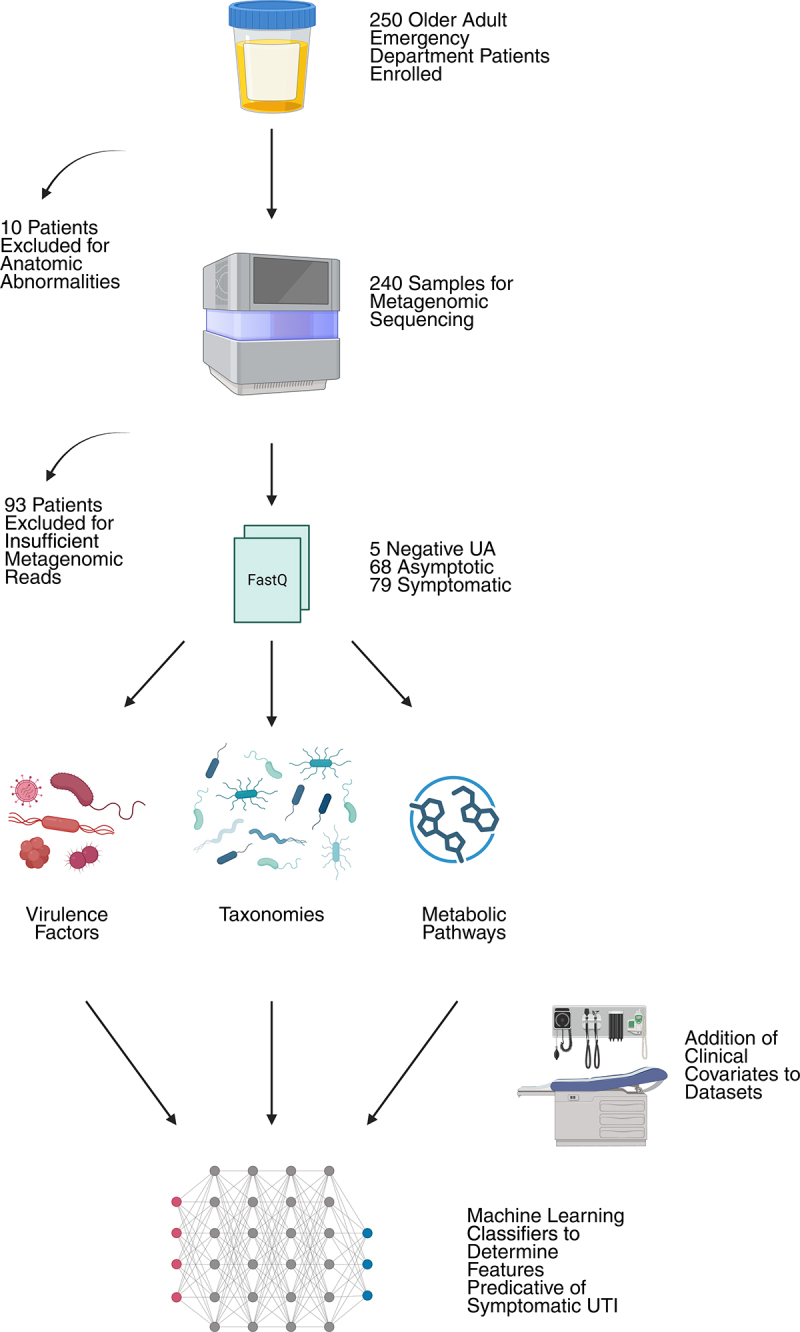
Study overview and analysis scheme.

The overall study schema is shown in Figure 1. Of 250 enrolled patients, 10 were excluded due to anatomic abnormality (suprapubic catheter, urostomy, etc.). An additional 93 were excluded for insufficient metagenomic reads. Although this represents a large portion of the cohort, this exclusion ensured that low abundance contaminants that could be introduced by handling and batch effects from different sequencing runs did not overly impact downstream analysis. Of the remaining cohort, 147 returned positive UAs with or without urinary symptoms while 5 had negative UAs, all without symptoms.

**Figure 2.**
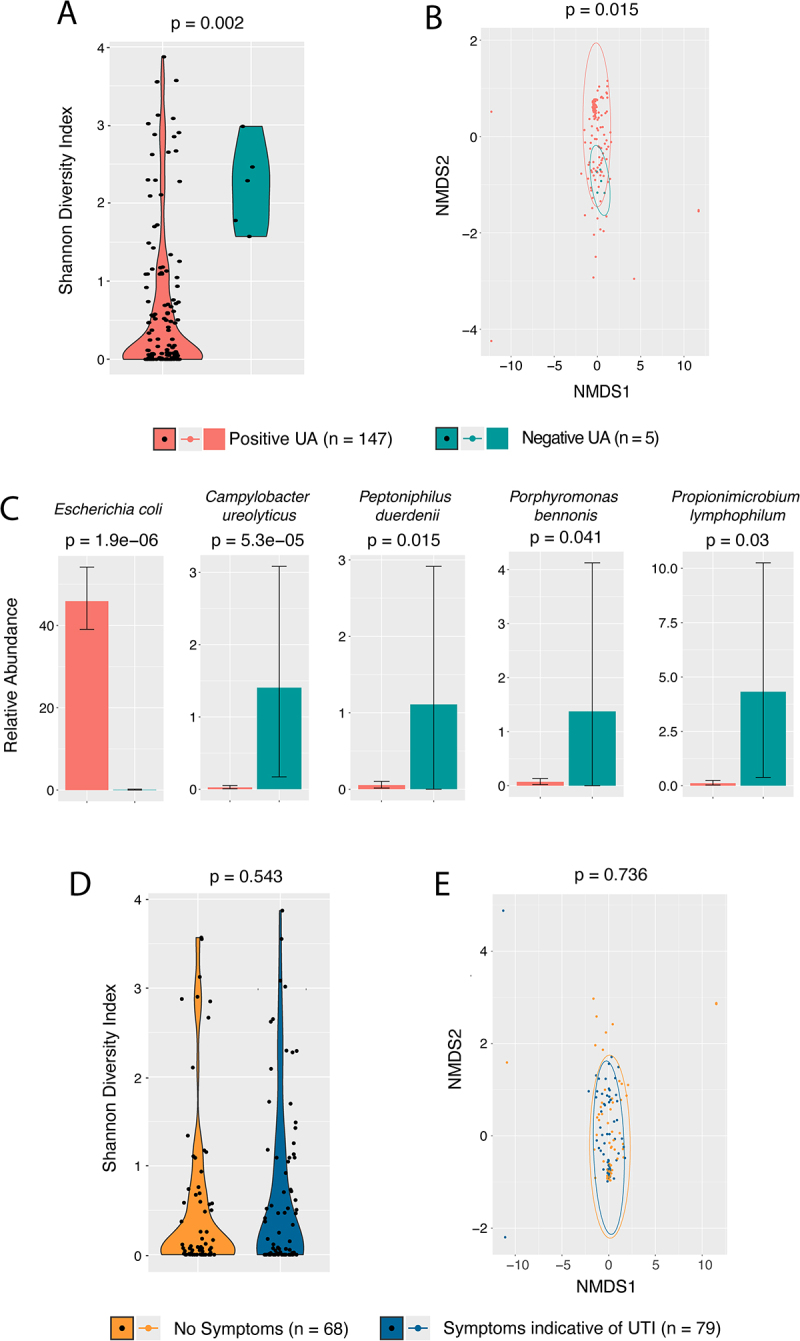
Community level microbiome metrics comparing positive UA versus negative UA, and asymptomatic vs symptomatic cases. (A) Subjects with a negative UA had significantly higher alpha diversity of their urinary microbiome (median Shannon index 2.286 for control versus 0.08633 for positive UA, *p* = 0.002 Mann-Whitney U-test). (B) NMDS ordination plot of positive UA and Negative UA microbiomes shows that the positive UA cohort had substantially more variation between samples compared to negative UA. (C) Differential abundance of taxa that vary significantly between negative UA and positive UA using Maaslin2. Shown is the mean and standard deviation of relative abundance of each taxa as well as p-value associated with coefficient in the model. *E. coli* represented a substantial portion of mapped microbial reads from those with positive UA compared to negative, but the relative abundance of some organisms was higher in the negative UA samples. (D) Shannon alpha diversity index did not vary across asymptomatic vs symptomatic cases (median Shannon index in 0.0819 versus 0.116, *p* = 0.543 Mann-Whitney U-test). (E) NMDS ordination plot of Bray-Curtis distance between asymptomatic vs symptomatic cases, significant overlap between the two states is seen.

When examining negative UA vs positive UA microbiomes, negative UA communities showed higher richness (number of species in each microbiome) (48.4 mean species negative UA versus 19.7 positive UA, *p* = 0.01 for ANOVA on richness), and evenness (similar abundance across all species detected) (mean Pielou’s evenness 0.59 negative UA and 0.3 positive UA, *p* = 0.018 for ANOVA on evenness). Alpha diversity, a combined metric reflecting richness and evenness within individual samples, as measured by Shannon index (Figure 2(A)), was significantly higher in negative UA microbiomes (median Shannon index 2.286 for negative UA versus 0.08633 in positive UA samples, *p* = 0.002 Mann-Whitney U-test). Examining beta diversity, a metric of how much microbiome compositions differ between groups, we found significant centroid distances in Non-metric MultiDimensional Scaling (NMDS) ordination plots (Figure 2(B)) between positive UA and negative UA groups (PERMANOVA on Bray-Curtis distance *p* = 0.0033), variances were not significantly different (ANOVA on beta-dispersion *p* = 0.778). Utilizing the Microbiome Multivariable Association with Linear Models (Maaslin2) package [32], we found that that positive UA samples contained more *Escherichia* (*p* = 1.9 ×10^−6^). Other organisms detected in much smaller amounts included *Campylobacter ureolyticus* (*p* = 5.3 ×10^−5^), *Peptoniphilus duerdenii* (*p* = 0.015), and *Propionibacterium lymphophilum* (*p* = 0.04) were higher in the negative UA samples (Figure 2(C)).

We next examined positive UA samples as symptomatic versus asymptomatic cases on the basis of signs and symptoms of UTI (68 categorized as asymptomatic, 79 as symptomatic). The baseline characteristics of symptomatic and asymptomatic patients were very well balanced with respect to demographics, recent antibiotic treatment, hospitalizations, primary residence, baseline incontinence of stool or urine, and dementia diagnoses (Table 1). The only medical history that varied between groups was a history of Chronic Obstructive Pulmonary Disease (COPD) (*p* = 0.009, chi-square). The association between UTI and COPD among older adults has been reported in the past [42]. Individuals with COPD are thought to be at increased risk of UTI due to treatment with anti-cholinergic medications used to manage COPD causing urinary retention [43]. We also examined if any features of the microscopic UA typically used to diagnose infection (white blood cells/hpf, red blood cells/hpf, presence of nitrites, qualitative presence of bacteria) varied across symptomatic and asymptomatic cases. There were no significant differences in any of these metrics when examined across symptomatic vs asymptomatic cases (Supplemental Figure 1).

**Figure 3.**
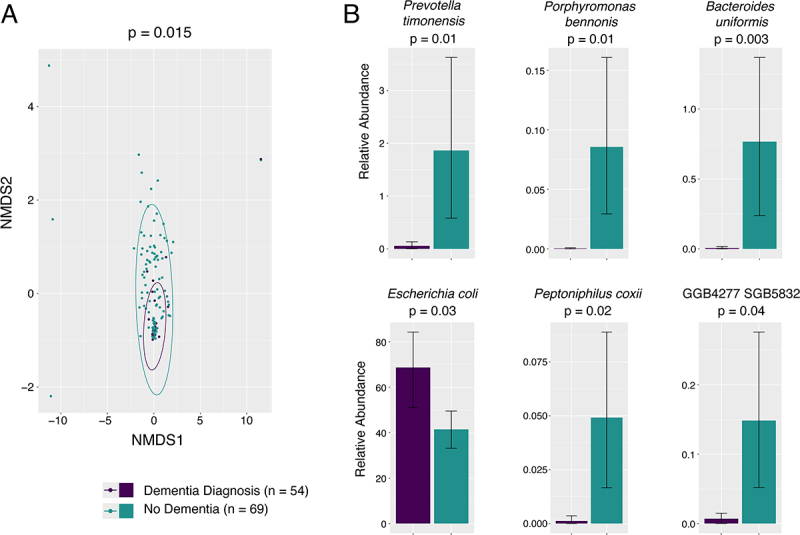
Urinary microbiome among subjects with dementia compared to those without dementia. (A) NMDS ordination plot of Bray-Curtis distance between urinary microbiomes among subjects with and without a diagnosis of dementia (PERMANOVA on centroid distances *p* = 0.015). (B) Mean relative abundance of taxa in urinary microbiomes found to be predictors of dementia diagnosis by Maaslin 2. Shown is mean and standard deviation of relative abundance and p-value of coefficients determined by Maaslin2. *E. coli* shows substantial higher relative abundance in individuals with dementia, other taxa were present at lower relative abundance and higher in individuals without dementia.

We next examined broad metrics of microbiome composition across these groups. Alpha diversity did not differ significantly between asymptomatic and symptomatic cases (median Shannon index in 0.0819 versus 0.116, *p* = 0.543 Mann-Whitney U-test) (Figure 2(D)). No significant difference in the centroid distances between asymptomatic and symptomatic cases was seen (PERMANOVA on Bray-Curtis distance *p* = 0.736) (Figure 2(E)). We also explored Bray-Curtis distance between microbiomes associated with clinical covariates of interest, including urinary catheterization within the prior three months, sex, living in the community versus residing within a nursing home or assisted living apartment, hospitalization or surgery within the last three months, presence of dementia, incontinence of stool or urine, and antibiotic treatment for any indication within the last three months, the complete results of which are listed in Table S1. Beta-diversity did not differ when examining microbiomes from male and female participants, which was also true in our previous study of the urinary microbiome of nursing home residents [44], and may reflect age-related changes in the microbiome that are shared among men and women. When comparing participants with and without a diagnosis of dementia, there were significant differences in beta diversity (PERMANOVA on Bray-Curtis *p* = 0.015, *n* = 123 without dementia, *n* = 24 with some dementia diagnosis), although the beta dispersion did vary significantly between these groups as well (ANOVA on beta-dispersion *p* = 0.0016) which could be somewhat responsible for this finding (Table S1). The NMDS ordination plot of Bray-Curtis distances between these two groups is shown in Figure 3(A). We also explored which taxa were associated with dementia diagnosis using Maaslin2. Six species associated with a diagnosis of dementia. *E. coli* was returned as having a positive association with the diagnosis of dementia (*p* = 0.003) while five other taxa present at lower relative abundance showed a negative association with dementia. Mean relative abundances of these taxa across the two groups and significance of the association are shown in Figure 3(B).

We did explore differences in clinical covariates among study participants with and without a diagnosis of dementia, such as age, recent urinary catheterization, hospitalization and prior treatment with antibiotics directed against UTI. These results are shown in Table 2. Patients with a diagnosis of dementia were older (*p* = 0.047, Mann-Whitney U test), but no other clinical covariate we hypothesized could contribute to urinary microbiome dysbiosis varied significantly differently across the groups, although there was a trend towards additional antibiotic treatment for UTI in the past (Chi-squared *p* = 0.096).

**Table 2.** Examination of clinical factors across patients with and without dementia.

	Any Dementia Diagnosis	No Dementia	p
n	24	123	
Age (median [IQR])	84.00 [77.00, 87.50]	79.00 [72.00, 86.00]	0.047*
Female (%)	20 (83.3)	97 (78.9)	0.826
White (%)	21 (87.5)	114 (92.7)	0.659
Black (%)	1 (4.2)	3 (2.4)	1
Other (%)	2 (8.3)	6 (4.9)	0.849
Hispanic (%)	1 (4.2)	5 (4.1)	1
Antibiotics within 90 Days (%)	11 (45.8)	43 (35.0)	0.436
Antibiotics for UTI specifically within 90 Days (%)	8 (33.3)	20 (16.3)	0.096
Urinary Catheterization within 90 Days (%)	4 (16.7)	12 (9.8)	0.525
Hospitalization within 90 Days (%)	9 (37.5)	41 (33.3)	0.874
Surgery within 90 Days (%)	4 (16.7)	20 (16.3)	1
Baseline Incontinence of Urine (%)	11 (45.8)	61 (49.6)	0.909
Baseline Incontinence of Stool (%)	3 (12.5)	16 (13.0)	1
Community Dwelling (%)	19 (79.2)	106 (86.2)	0.57
History of Chronic Kidney Disease (%)	10 (41.7)	40 (32.5)	0.529

**Figure 4.**
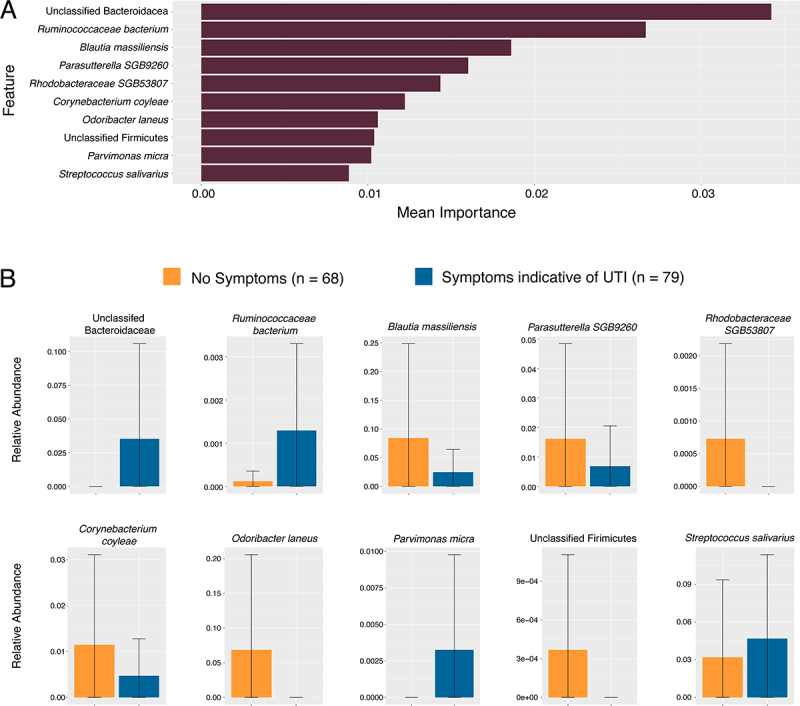
Top Ten features of TabNet model on relative abundance of taxonomies and clinical covariates. (A) Graphed mean importance of each feature in the model. (B) Mean relative abundance of top 10 most important taxa in predicting asymptomatic versus symptomatic cases in the TabNet model, error bars represent standard deviation of the mean. No formal statistical tests are performed on these features aside from TabNet analysis and as such no p-values are reported.

**Figure 5.**
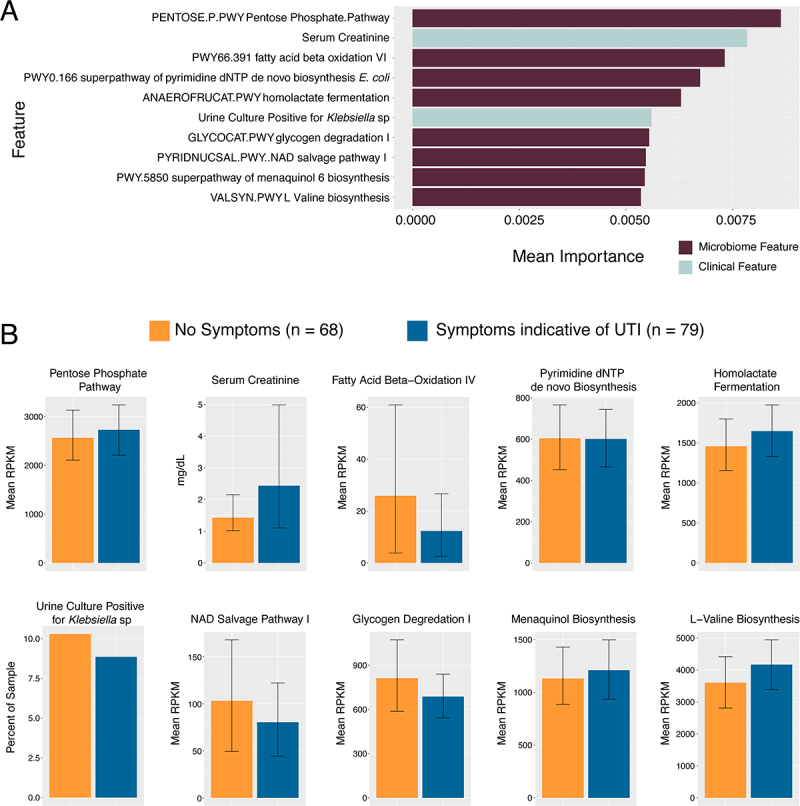
Top Ten features of TabNet model on RPKM of bacterial metabolic pathways and clinical covariates. (A) Graphed mean importance of each feature in the model. (B) Graphed mean RPKM and standard deviation of reads mapped to established metabolic pathways split by asymptomatic versus symptomatic cases. For serum creatinine, this is measured mg/dL abstracted from clinical data. For positive urine culture for Klebsiella species, graphed is the percent positive samples from asymptomatic vs symptomatic cases respectively. No formal statistical tests are performed on these features aside from TabNet analysis and as such no p-values are reported.

**Figure 6.**
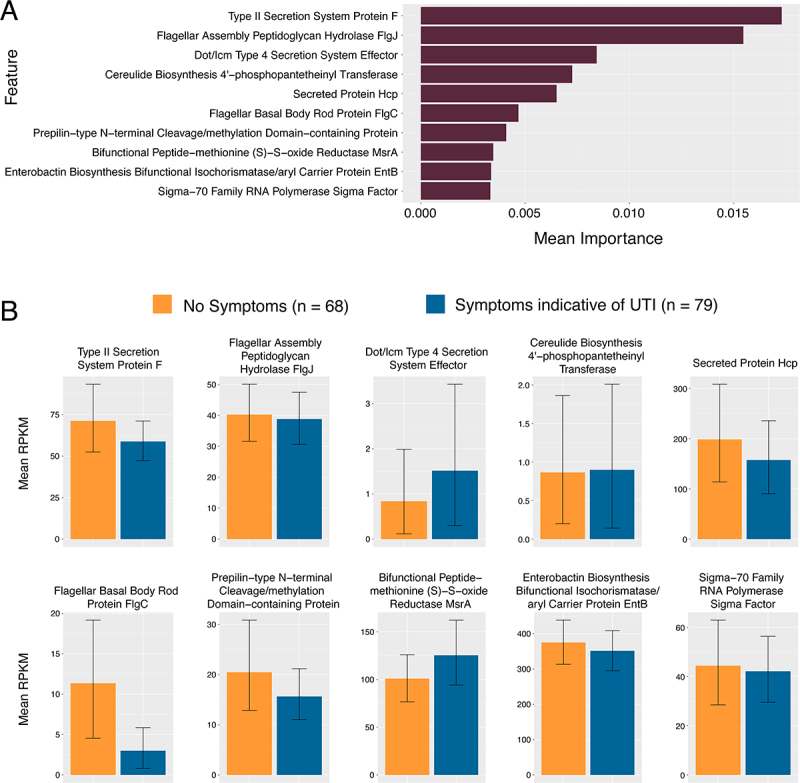
Top Ten features of TabNet model on RPKM of known virulence factor genes and clinical covariates. (A) Graphed mean importance of each feature in the model. (B) Mean RPKM and standard deviation of reads that mapped to known virulence factors split between asymptomatic versus symptomatic cases. No formal statistical tests are performed on these features aside from TabNet analysis and as such no p-values are reported.

An advantage of metagenomic sequencing is being able to analyze microbiome features beyond taxonomy. Thus, we generated datasets of relative abundance of genes within known metabolic pathways [27] and relative abundance of known virulence factors by counting reads aligned against the VFDB database of protein sequences associated with known virulence factors [28]. We trained machine-learning based classifier models on three multimodal datasets: available clinical data (without specific signs and symptoms of UTI used to classify cases) combined with either relative abundance of taxa, RPKM of metabolic pathways, or RPKM of known virulence factors. The neural network-based TabNet showed the most consistent results across all performance metrics and datasets. Some penalized regression models such as LASSO and ridge regression showed higher mean F1 scores, but at the cost of specificity (Table S2). Most of the models we tested return ranked importance of which features of the modeled datasets contributed most to the classification outputs (symptomatic versus asymptomatic). In most models, microbiome features (taxa abundance, metabolic pathways, or virulence factors) were ranked as more important than non-symptom clinical inputs. We selected TabNet as our final model due to its consistent performance across all datasets in this study. Compared to other classifiers, TabNet achieved the most balanced trade-off between sensitivity (recall) and specificity. Figures 4–6 (top panels) show the ranked importance from the TabNet models.

Examining the important features in the TabNet model built on relative abundance of different taxa, the relative abundance of a species of an unclassified *Bacteroidaceae* (mean relative abundance 0% in asymptomatic versus 0.035% in symptomatic), was the most important feature, followed by the abundance of *Ruminococcaceae bacterium*, with higher relative abundance in our symptomatic group (mean relative abundance 0.00012% asymptomatic versus 0.0013% in symptomatic). The abundance of *Blautia massilensis (*mean relative abundance 0.085% versus 0.025%) and a *Parasutterlla* (mean relative abundance 0.016% versus 0.00012%) taxa were the next two most important features and showed higher abundance in urinary microbiomes from individuals classified as asymptomatic (Figure 4). In the model built on metabolic pathway abundance, summarized in Figure 5, the two most important features were the abundance of the Pentose-phosphate pathway (mean RPKM 2730 in symptomatic versus 2567 in asymptomatic), and a clinical covariate, the level of serum creatinine, both higher in symptomatic patients (Figure 5). The pathways for homolactate fermentation, and menaquinone biosynthesis were also higher in microbiomes of symptomatic patients (mean RPKM 1647.5 versus 1131.5 and mean RPKM 1210.5 versus 1131.5 respectively). Pathways for fatty acid beta-oxidation, nicotinamide (NAD) salvage, and glycogen degradation were more abundant in asymptomatic cases (mean RPKM 25.3 versus 12.3, 103 vs 80, and 812.5 vs 687.5 respectively). Another clinical covariate, a positive urine culture for *Klebsiella* was slightly higher in asymptomatic cases. In the model built on relative abundance of virulence factors, summarized in Figure 6, the most important features were a type II secretion protein (genbank ID WP_016352169) (mean RPKM 58.1 in symptomatic versus 71.8 in asymptomatic), a component of flagellum assembly (genbank ID WP_015444575) (mean RPKM 38.9 versus 40.3) and a type IV secretion system effector protein (Genbank ID WP_010948683) (mean RPKM 1.5 vs 0.83).

## Discussion

There appears to be a clear distinction in our cohort between the urinary microbiome from patients with a positive microscopic UA and those whose UA do not show any findings consistent with infection (negative UA). These include much higher alpha diversity, higher richness, and evenness for negative UA and clear differences in beta diversity between negative and positive UA. This finding is consistent with studies of the microbiome in the gastrointestinal tract [45], which show that in general, a more diverse microbiome is reflective of a healthy microbiome; in this way a UA with findings consistent with infection and lower in microbial compositional diversity could be considered a dysbiotic or unhealthy state. The presence of diverse commensal organisms could potentially be harnessed as testing for a “healthy” urinary microbiome and could be targeted in future studies to determine if they perform some active function in preventing pathogen colonization. Alternatively, the presence of inflammation within the urinary tract could also drive dysbiosis. Previous work has shown that in both ASB and UTI, there are higher abundances of proteins associated with neutrophil activation, degranulation, and chemotaxis in the urine of individuals with evidence of infection [46].

Of particular interest is the overwhelming presence of *E. coli* in those with positive UA, suggesting this organism is much more common in subjects with a positive UA, whether they have symptoms or not. *C. ureolyticus*, *P. duredeni*, and *P. lymphophilum* were more abundant in negative UA microbiomes. These organisms have been variously reported to be potential human pathogens [47–49], but their presence here in the urine of patients with negative UAs indicates they may be beneficial commensal organisms. Alternatively, they may become pathogenic in the setting of dysbiosis or specific perturbation of commensal community structure. Thus, normal colonization of specific bacteria within a healthy urinary microbiome may exist along a continuum wherein dysbiosis of specific species may precede ASB and UTI and symptoms present in context with immune activation and an altered uromicrobiome.

An unexpected result was a difference in beta-diversity of the urinary microbiome between patients with dementia versus those without. Although beta-dispersion did vary between these groups, PERMANOVA is considered to be a test that is least likely to be affected by heterogeneity in beta-dispersion between groups [50]. When examining what features were associated with a dementia diagnosis using Maaslin2, the potential pathogen, *E. coli*, was detected more frequently and in higher abundance in those with a dementia diagnosis, while higher abundances of *Prevotella timonensis, Porphyromonas bennonis, Bacteriodes uniformis, Peptoniphilus coxii*, and an uncharacterized taxa were negatively associated with dementia. While some of these organisms may be opportunistic pathogens [51–53], their presence at relatively low abundance suggests they are likely commensals in this context, and overgrowth of *E. coli* may represent dysbiosis among individuals with dementia. We did explore non-symptom clinical factors among patients with dementia that could potentially explain the observed dysbiosis such as recent antibiotic treatment, hospitalization, urinary catheterization, or residing in the community versus a nursing home. Although patients with dementia were older (median age 84 with dementia, 79 without, *p* = 0.047 Mann-Whitney U test), no other clinical covariate was significantly associated with dementia (Table 2). How dementia may be driving this dysbiosis or vice versa could be investigated in the future.

On initial examination, the most striking feature of our cohort of subjects with positive UAs is the very high number of asymptomatic cases (68 cases or 46%). These patients should not have had urinalysis obtained at all if clinical guidelines were strictly adhered to. The microbial community of individuals with a positive UA was remarkably similar whether they had signs and symptoms of UTI or not. No diversity metrics showed significant differences. We have used machine learning-based classifiers to explore multimodal microbiome data to find meaningful microbiome features associated with clinical conditions or outcomes in the past and applied it to this dataset [54,55]. We employed a wide array of popular machine learning-based classifier algorithms, of these, the neural-network based TabNet model performed most consistently across metrics. TabNet’s architecture supports instance-specific feature selection through its sequential attention mechanism. Unlike Lasso, Ridge, or SVM, which use a fixed set of features across all samples, TabNet adjusts the relevance of features for each case. This flexibility is particularly important in microbiome-based classification, where patient-specific microbial signatures often vary substantially. As shown in Table S2, TabNet has the most consistent performance metrics with superior specificity, consistent recall, sample-level feature selection, and low variance across seeds.

Although we hypothesized that microbiome features would reliably distinguish symptomatic versus asymptomatic patients and that these features could subsequently be examined for use as enhanced diagnostics for true UTI, we acknowledge that the performance of these models is not reliable enough to be used in clinical testing to discriminate between potential ASB and UTI. The high number of asymptomatic cases and the modest performance of our in-depth microbiome analysis highlight that antibiotic stewardship campaigns that focus on education and adherence to guidelines remain the most likely interventions to reduce inappropriate testing and antibiotic treatment. However, the high importance of microbiome features in our models provides biological insight into the importance of the urinary microbiome in these two states, and we believe some broad themes emerge.

In our model built on taxonomies, we observed that the most important feature was the relative abundance of an uncharacterized *Bacteroidaceae* strain, which was more abundant in microbiomes classified as symptomatic. This family is typically anaerobic and associated with the gut microbiome. Its presence here may indicate ascending colonization of microbiota from the gut which is suggested to be the source of some uropathogens [56]. Another taxa that showed higher abundance in symptomatic microbiomes was a strain of *Ruminococcaceae*, which is a potential pathobiont that has been associated with the urinary microbiome of individuals with bladder cancer [57] and was positively correlated with lower urinary tract symptoms in men [58]. Another taxa with higher abundance in microbiomes classified as asymptomatic include a *Parasutterella* species, a taxa that is more typically observed in the gut microbiome [59], but interestingly, is depleted in individuals who suffer from recurrent UTIs [56], suggesting it maybe a protective commensal. *B. massilensis* was also more abundant among asymptomatic patients, members of this family and genus have been negatively associated with urinary symptoms in men [58], suggesting it may be another protective commensal.

When examining the model built on relative abundance of microbial metabolic pathways and non-symptom clinical features, important features may be suggestive of a role for specific patho-adaptations in eliciting symptoms. Features more abundant in microbiomes from symptomatic patients included the pentose phosphate pathway, homolactate fermentation, and menaquinol biosynthesis. Considering these in the context of infection, the pentose phosphate pathway in *E. coli* is involved in generating reducing potential and building blocks for biomolecules [60] and is upregulated in *E. coli* in the setting of UTI [61]. Oxygen tension in urine can be quite low and it is likely that anaerobic fermentation (such as homolactate) pathways are important virulence factors for UPEC [62]. Menaquinone is specifically involved in anaerobic metabolism and UPEC mutants in this pathway show decreased fitness in models of urinary tract infection [63]. Important microbial pathways with higher abundance in microbiomes classified as asymptomatic included fatty acid beta oxidation, NAD salvage pathway, and glycogen degradation. Fatty acid biosynthesis, as opposed to degradation, has been observed to be upregulated in *E. coli* isolated from human UTI specimens [64]. *E. coli* seems to rely on nucleotide salvage for planktonic growth in urine, but requires *de novo* synthesis during invasive infection [60]. Glycogen metabolism was seen to be important in a mouse model of *E. coli* intestinal colonization [65], but utilization of other carbon sources seems to be more important in causing UTI [66]. Overall, pathways associated with colonization show higher abundance in microbiomes classified as asymptomatic, whereas those that seem to be important in rapid growth are more abundant in symptomatic UTI microbiomes. Indeed, the ability to grow rapidly in the relatively anaerobic host environment may be the decisive factor in causing infection [67] as opposed to simply colonizing the urinary tract. Of note, two clinical variables appeared among the top ten features in this model, including a higher serum creatinine in symptomatic individuals, which may reflect decreased kidney function in individuals suffering from UTI, and the percentage of patients with positive urine cultures for *Klebsiella*, suggesting that this organism may more commonly be associated with asymptomatic colonization.

When examining the model built on relative abundance of virulence factors and clinical features, two important themes were secretion systems and bacterial flagellum components. Genes associated with flagellum assembly (flgJ and flgC) were more abundant in asymptomatic microbiomes. The flagellum is a structure involved in bacterial motility, and in a mouse model of cystitis, this was seen to be important in persistence in the bladder [68]. Type II and Type IV secretion systems are ubiquitous among Gram negative pathogens and may have a role in the pathogenesis of UPEC [69]. Interestingly, the type II secretion system component gspF was more abundant in asymptomatic microbiomes where as a Type IV secretion effector was more abundant in symptomatic microbiomes (mean RPKM 1.5) versus asymptomatic (mean RPKM 0.84). These systems are pathogenicity factors for a wide range of Gram-negative pathogens [70], but their role in UTI has not been established.

It is important to note that the classifiers used here return feature importance in predicting outcomes as defined by our clinical and laboratory criteria. However, they do not inherently return the direction of the association (e.g. whether a higher or lower abundance, presence or absence of a feature is predictive) or a traditional statistical confidence or p-value associated with feature importance. Neural networks can consider features in combination; so the abundance in each individual sample may only be predictive as part of a combination of other feature patterns considered in the model. In our discussion, we have simplified the analysis of predictive features by examining their abundance in one state versus another to attempt to make inferences regarding biologic processes which may underly the development of symptomatic UTI. These features should be considered together as part of a pattern distinguishing microbiomes from asymptomatic and symptomatic patients and we have tried to highlight unifying themes within results.

## Strengths and weaknesses

This was a well-balanced cohort that incorporated clinical covariates and microbiome features into our analysis and showed significant differences between balanced groups (symptomatic versus asymptomatic, dementia versus no dementia). Strengths also included microbiome measurements taken from point-of-care samples, inclusion of laboratory testing, and detailed surveys of current signs and symptoms for as accurate of a distinction between UTI and ASB diagnosis as possible on the basis of all available data in the acute care setting. We also were able to include patients who lacked capacity to consent, a group for which clinicians face difficulty in obtaining history of UTI symptoms and represent a greater diagnostic challenge. Although our final F1 scores from machine learning models were modest, all models trended towards positive ( > 0.5) predictions.

A weakness of this study includes its relatively small final cohort (68 asymptomatic patients, 79 with symptoms, and 5 patients with negative UA). As such, results of our analysis of negative UAs may not be widely generalizable as this is based on the small sample size of five individuals. Many patients/samples had to be discarded because of very low metagenomic content, which was necessary to ensure robust microbiome data for analysis. In microbiome analysis of low biomass sites like urine and airway, it is possible that contamination by handling or introduced by lab equipment can bias results [71]. Although we did not perform negative control sequencing samples, we did not detect as significant any taxa commonly associated with skin or environmental microbiome. Also, issues with contamination can be avoided with higher biomass inputs, which we effectively instituted by using a minimum metagenomic sequencing cut off [72]. Analyzing samples with minimum metagenomic content also helps prevent bias from different sequencing batches [73].

Another limitation was the use of convenience samples, which had to be evaluated and ready for research staff to obtain during regular business hours. It is possible that some unmeasured bias for certain patient types could be introduced by this practice and the results may not be generalizable across patients presenting at any time. Nonetheless, our measured demographic factors were well balanced across asymptomatic and symptomatic patients. Another weakness and potential explanation for our modest F1 scores may be that the patients who may have only had nonspecific symptoms, such as fever and altered mental status, may have had an alternative final diagnosis that was not captured by our 14-day chart review. It is also possible that individuals with dementia would be considered asymptomatic more frequently as they may have limited ability to report symptoms such as dysuria. However, our cohorts were balanced with respect to dementia diagnosis, so we do not believe that there was a bias towards classifying these patients as asymptomatic. Non-specific signs and symptoms like fever or altered mental status in the setting of a positive UA would usually be considered diagnostic and 84% of such cases in our cohort were indeed treated with antibiotics directed against UTI specifically. The IDSA guidelines do recommend that additional testing be performed for alternative explanations in such cases before initiating treatment for UTI [13], but our data does not capture what, if any, the results of those additional tests might be. If an alternative explanation was responsible for a patient’s symptoms, some cases could be later classified more accurately as asymptomatic for machine learning model training. Despite these challenges, we were able to describe significant community level differences between microbiomes present in positive versus negative UAs, as well as unexpected community differences in patients with and without dementia. Our machine-learning based analysis did identify microbiome features primarily as predictive of asymptomatic versus symptomatic cases, suggesting that there are features within uromicrobiome that are associated with symptoms in UTI.

## Conclusions

This metagenomic analysis of older adult ED patients with suspected UTI highlights the continued difficulties of diagnosing and treating older adult patients for UTI, especially in the acute care setting. Nearly half of our participants with a positive UA did not have signs and symptoms that would qualify for testing for UTI based on IDSA and Massachusetts Department of Public Health guidelines (and thus should not have been tested in the first place). While this can be regarded as a failure of strict guideline adherence on the one hand, on the other hand, the results of these asymptomatic UAs may give us insight about a previously underappreciated biologic process: the transition from a healthy to a dysbiotic uromicroboime and further, the transition from ASB to true clinical infection. Indeed, clear definitions between these transition points and states are still undefined. Machine learning-based methods are becoming more prevalent in the analyses of complex microbiome data, and we have attempted to apply it here to divulge insight into what distinguishes ASB from UTI from a clinical perspective based on acute care setting information. Our analyses suggest some functional uromicrobiome characteristics (genes favoring colonization activities versus virulence activities) may aid in distinguishing these two states and may offer clues into the biologic mechanisms that result in physiologic symptoms of UTI.

## Supplementary Material

Methods supplement.docx

Figure_S1.tif

## Data Availability

Sequence files and metadata are available in the NCBI’s Short Read Archive (SRA) under the project number PRJNA1208988 by following the following link: https://www.ncbi.nlm.nih.gov/bioproject?LinkName=sra_bioproject&from_uid=36843498
