# Supplementary material for: The urinary microbiome distinguishes symptomatic urinary tract infection from asymptomatic older adult patients presenting to the emergency department
Source: Virulence. 2025 Aug 9;16(1):2546063. doi: 10.1080/21505594.2025.2546063 (PMC12351761; doi:10.1080/21505594.2025.2546063)
Supplement: Methods supplement.docx [file KVIR_A_2546063_SM9462.docx]

Least Absolute Shrinkage and Selection Operator(LASSO)and Ridge regression are variations on logistic regression known as penalized regression models that work by shrinking the size of coefficients in a regression model to avoid overfitting and multicollinearity. This process helps to identify a smaller number of predictors that can be used to build a reliable model with complex starting data. Elastic Net is a model that combines the penalties of both LASSO and Ridge regression. Support Vector Machine (SVM) regression uses a transformation or kernel function to transform data to reveal discriminatory features. Random Forrest Classifier (RFC) is a combination of decision trees such that each tree depends on the values of randomly sampled variables independently and with the same distribution for all trees in the forest. A prediction from an RFC model represents the aggregate or consensus of commonly hundreds to tens of thousands of trees. RFCs are becoming more common in complex biologic studies such as microbiome investigations. XGBoost is based on a similar underlying architecture as RFC, that is, decision trees. However, it employs a regularized model framework to prevent overfitting and supports a variety of objective functions, including weighted classification and ranking, demonstrating its proficiency in managing tabular data. These enhancements aggregate and “boost” the signals of “shallow” or weak decision trees in iterative training, thereby enhancing signal to noise while attempting to minimize errors. We employed XGBoost Default. TabNet is a neural network-based algorithm designed specifically for tabular data that effectively utilizes sequential attention mechanisms to identify and leverage the most salient features at each decision point, enhancing learning efficiency by focusing its capacity on the most significant features. In essence, the algorithm is designed to learn the most important features in the model, or those that have the highest ability to discriminate between outcomes and adjusts weighting for those features. All models were dynamically processed using four NVIDIA 3090 GPUs within an Ubuntu 20.04 environment, utilizing Python 3.10.13 along with PyTorch 2.2.1, PyTorch-TabNet 4.1.0, XGBoost 2.0.3, and Optuna 3.5.0 for hyperparameter tuning.

We implemented a stratified 70:30 train-test split to mitigate potential bias and ensure reproducibility while preserving class balance. We utilized an internal 80:20 validation split within the training data for hyperparameter tuning through Optuna optimizing for log loss. Optuna automates the search for optimal hyperparameters, streamlining the model optimization process through Bayesian optimization, Tree-structured Parzen Estimator (TPE), or other algorithms, thereby enhancing the efficiency of the optimization process. This process helped ensure probabilistic calibration while reducing the risk of overfitting. All models were evaluated across ten random seeds, each generated deterministically using MD5-hashed experiment identifiers. We averaged performance metrics across these repetitions and reported them as the median, allowing us to assess generalization performance and stability under random initialization and data shuffling. For these models, we used the Message-Digest Algorithm 5 (MD5) to generate 10 random seeds for each of the three datasets, utilizing our data experiment IDs for this purpose. For median performance metrics across the 10 seeds of these models, see Table S2.

.
