# Supplementary figures and images for: The urinary microbiome distinguishes symptomatic urinary tract infection from asymptomatic older adult patients presenting to the emergency department
Source: Virulence. 2025 Aug 9;16(1):2546063. doi: 10.1080/21505594.2025.2546063 (PMC12351761; doi:10.1080/21505594.2025.2546063)

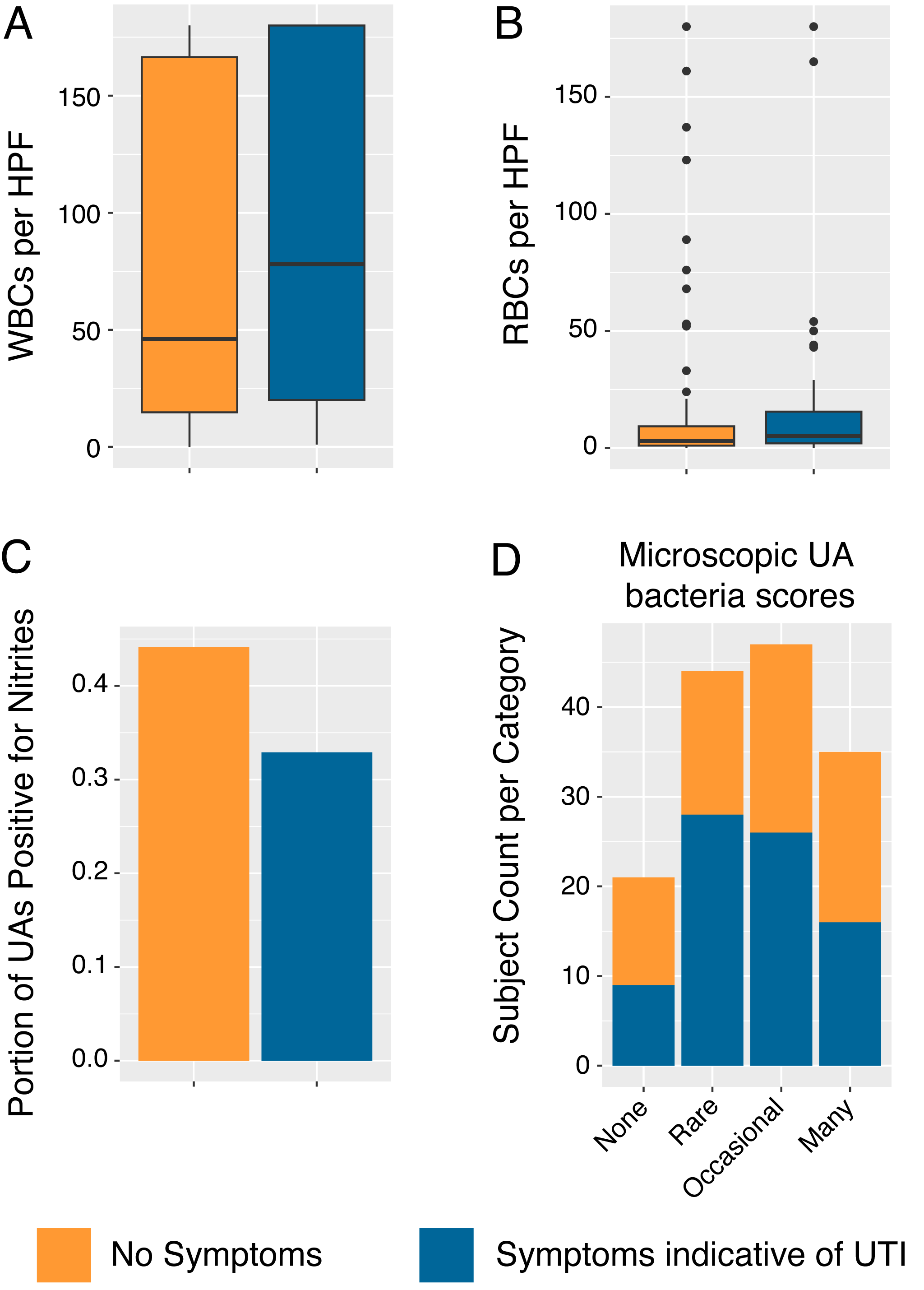

Supplement: Figure_S1.tif [file KVIR_A_2546063_SM9461.tif]
